# Quantitative D-Dimer in Suspected Cerebral Venous Thrombosis: Diagnostic Performance and Clinical–Radiological Associations

**DOI:** 10.3390/jcm15208051

**Published:** 2026-10-16

**Authors:** Fatma Yılmaz Can, Meryem Tuba Sönmez, Aynur Turan

**Affiliations:** 1 Department of Neurology, Etlik City Hospital, 06170 Ankara, Türkiye; mtubas@gmail.com; 2 Department of Radiology, Etlik City Hospital, 06170 Ankara, Türkiye; aturanrad@gmail.com

**Keywords:** cerebral venous thrombosis, diagnostic accuracy, magnetic resonance imaging, quantitative D-dimer

## Abstract

**Objective:** This study aimed to evaluate the diagnostic accuracy of quantitative D-dimer levels in patients investigated for suspected cerebral venous thrombosis (CVT) and their relationship with clinical and radiological characteristics in patients diagnosed with CVT. **Methods:** Based on neuroimaging findings, 97 consecutive patients investigated for suspected CVT were divided into CVT (*n* = 54) and CVT-negative *(n* = 43) groups. Quantitative D-dimer levels were compared between the groups using a cutoff value of 0.5 mg/L. The sensitivity, specificity, positive and negative predictive values, likelihood ratios, and area under the ROC curve (AUC) of elevated quantitative D-dimer values for the diagnosis of CVT were calculated. In patients with CVT, correlations between quantitative D-dimer levels and sex, age, symptom duration, presence of neurological deficits, the National Institutes of Health Stroke Scale (NIHSS) score, number of thrombosed sinuses, and presence of parenchymal damage were examined. **Results:** Quantitative D-dimer levels were elevated in 90.7% of the CVT group and 4.7% of the CVT-negative group (*p* < 0.001). Quantitative D-dimer had a sensitivity of 90.74%, specificity of 95.35%, positive predictive value of 96.08%, negative predictive value of 89.13%, and AUC = 0.967 for CVT. Quantitative D-dimer levels were inversely correlated with symptom duration (*p* = 0.027), whereas no statistically significant correlations were found with sex (*p* = 0.443), age (*p* = 0.696), the presence of neurological deficits (*p* > 0.05), NIHSS score (*p* = 0.079), the number of thrombosed sinuses (*p* = 0.627), and the presence of parenchymal lesions (*p* > 0.05). **Conclusions:** Elevated quantitative D-dimer levels can support the clinical decision-making process in distinguishing CVT. Levels decrease as symptom duration increases.

## 1. Introduction

Cerebral venous thrombosis (CVT) is a rare form of venous thromboembolism. Unlike arterial stroke, it is more common in young people and is more strongly associated with young women. It accounts for 0.5–3% of all strokes in the adult population, with an incidence of 1.3 per 100.000 people [1,2]. Its clinical presentation varies widely and may include headache, vomiting, seizures, blurred vision, double vision, muscle weakness, sensory disturbances, and varying degrees of altered consciousness. Papilledema is particularly evident in cases accompanied by increased intracranial pressure. Pregnancy and the postpartum period, oral contraceptive use, malignancy, infections, autoimmune and vasculitic diseases, and thrombophilic and hematological conditions are the main risk factors associated with CVT [3]. The diagnosis of CVT is primarily based on radiological imaging [4]. Depending on the circumstances, computed tomography (CT) and CT venography (CTV) with or without contrast, magnetic resonance imaging (MRI), and MR venography (MRV) are used for diagnosis [3]. However, physiological variations in venous anatomy make CVT difficult to diagnose. In particular, congenital stenosis or atresia of the transverse sinus and filling defects due to arachnoid granulation tissue may cause diagnostic uncertainty [5]. Changes in MRI signal characteristics with thrombus age may also complicate image interpretation. In the acute phase (0–5 days after the onset of clinical symptoms), the thrombus appears isointense to brain tissue on T1-weighted images (T1-WI) and hypointense on T2-weighted images (T2-WI) due to the abundance of deoxyhemoglobin. In the subacute phase (between 6 and 15 days after onset), the venous clot becomes more conspicuous; the signal is hyperintense on both T1- and T2-WI due to the accumulation of methemoglobin within the venous thrombus. In the chronic phase (two to four weeks after onset), the clot appears heterogeneous, showing isointensity on T1-WI and variable signal intensity (iso- or hyperintensity) on T2-WI, owing to the presence of deoxyhemoglobin and methemoglobin components. Consequently, signal changes depending on the age of the thrombus make diagnosis even more difficult [6]. In addition, idiopathic intracranial hypertension (IIH) may be difficult to distinguish from CVT because of shared clinical and radiological features, including headache, papilledema, cranial nerve involvement, and transverse sinus stenosis. The fact that CVT may present with headache alone, without focal neurological deficits, further complicates diagnostic assessment. To put it more clearly, 70.6% of SVTs present with isolated headache without focal neurological deficits [7]. Conversely, early treatment-related recanalization in patients with CVT has been associated with both early regression and a reduced risk of lesion enlargement [8]. It has been reported that delays in diagnosis and treatment may cause permanent disability or even death in patients with CVT [4]. Therefore, early recognition of suspected CVT and prompt initiation of appropriate treatment are essential.

Recent studies have emphasized the importance of elevated quantitative D-dimer levels alongside neuroimaging for early and accurate diagnosis [3]. Quantitative D-dimer is a degradation product of fibrinolysis and forms part of the vital balance between clot formation and dissolution in homeostasis [9]. Elevated serum quantitative D-dimer levels indicate increased fibrin degradation products, and D-dimer has been quantitatively measurable since the 1970s. It is considered a rapid and reliable bedside (point of care) diagnostic marker for many thrombotic diseases [10,11]. However, quantitative D-dimer is not elevated exclusively in thromboembolism; it is also an acute-phase reactant [12]. Its elevation in conditions such as inflammatory diseases, infections, trauma, and sepsis may create uncertainty regarding the underlying etiology [13]. Despite these considerations, low quantitative D-dimer levels are used as an adjunctive diagnostic tool to exclude thromboembolic conditions such as deep vein thrombosis and pulmonary thromboembolism because of their high negative predictive value [14]. More recently, on the premise that levels may also rise in CVT, quantitative D-dimer testing has been recommended as an additional adjunctive investigation in the differential diagnosis. Among emergency department patients with MRI/MRV findings equivocal for CVT, quantitative D-dimer levels were abnormally elevated in 94.1% of cases [7]. Another similar study found that elevated D-dimer levels had a high negative predictive value for excluding CVT in patients with isolated headache [10].

The introduction of advanced neuroimaging techniques has substantially reduced the diagnostic challenges associated with CVT. Nevertheless, limited access to specialized centers, the absence of a clinical presentation specific to CVT, physiological variations in cerebral venous anatomy, and the ability of IIH to closely mimic CVT remain diagnostic challenges. Additionally, while the diagnosis of CVT relies primarily on neuroimaging, other challenges include the lack of clear algorithms regarding the use of imaging, the absence of markers for rapid diagnosis, and the fact that the vast majority of patients present with isolated headache. Another important factor is that the majority of CVT patients consist of vulnerable individuals, such as young people, pregnant women, postpartum patients, and those with malignancies.

Thus, simple and reliable bedside tests are needed alongside neuroradiological imaging for the rapid diagnosis and treatment of CVT. This study was designed to use real-world data to investigate the diagnostic value of quantitative D-dimer levels for the rapid and accurate diagnosis of CVT and their relationship with radiological and clinical findings.

## 2. Materials and Methods

### 2.1. Study Design

This retrospective, single-center, observational diagnostic accuracy study was conducted in the Department of Neurology at Ankara Etlik City Hospital, a tertiary care center.

### 2.2. Study Population

The records of patients aged 18–72 years who were investigated for suspected CVT between November 2022 and June 2026 were retrospectively reviewed. The principal symptoms and signs prompting clinical suspicion of CVT were new-onset severe headaches or severe headaches that had changed in character, focal neurological deficits, altered consciousness, epileptic seizures, and papilledema on funduscopic examination. Pregnancy, the postpartum period, oral contraceptive use, and known neoplastic, vasculitic, or thrombophilic diseases were considered risk factors for CVT.

The records of 178 patients were reviewed during the study period. Patients with missing quantitative D-dimer test results, insufficient clinical information, or severe infections such as sepsis, meningitis, or encephalitis were not included in the study. Patients with a symptom duration exceeding 30 days were excluded because this could lead to false-negative results [14]. After the exclusion of 81 patients, the study continued with 97 patients.

Clinical assessments were performed by two neurologists with 15 years of experience in headache and stroke. Demographic characteristics, clinical and neurological examination findings, radiological characteristics, quantitative D-dimer levels, and the interval between symptom onset and D-dimer measurement were obtained from patient records.

### 2.3. Quantitative D-Dimer Measurement

Blood samples for quantitative D-dimer measurement were collected before neuroimaging and intravenous contrast administration. Samples were collected in tubes containing 2.7 mL of sodium citrate. Quantitative D-dimer levels were measured in citrated plasma using the Tina-quant D-Dimer Gen.2 latex particle-enhanced immunoturbidimetric assay on the cobas t 711 coagulation analyzer (Roche Diagnostics, Mannheim, Germany). Results were expressed in fibrinogen-equivalent units (FEU; mg/L). The manufacturer-stated expected value was <0.5 mg/L FEU. According to the manufacturer’s performance data, the intermediate precision coefficient of variation was 4.4% at a D-dimer concentration of 0.494 mg/L FEU (0.494 µg FEU/mL). Calibration was performed using the D-DI2 Cal Set according to the manufacturer’s instructions. A threshold of 0.5 mg/L, based on the laboratory reference value, was adopted as the prespecified cutoff. D-dimer values ≤ 0.5 mg/L were classified as normal, and values > 0.5 mg/L were classified as elevated [15].

### 2.4. Assessment of Radiological Data

Cranial CT was the initial neuroimaging examination in patients without contraindications. All patients included in the study underwent brain MRI and MRV to assess the cerebral venous system. The time-of-flight (TOF) technique was used for noncontrast MRV examinations. When considered necessary, the assessment was supplemented with gadolinium-enhanced brain MRI and/or contrast-enhanced MRV. The need for advanced contrast-enhanced imaging was determined by the neurologist and neuroradiologist on the basis of clinical and radiological findings. Contrast-enhanced MRV was performed on patients with sinus hypoplasia, atresia, or granulation tissue to ensure an accurate diagnosis. Of the 97 patients included in the study, 42 (43.3%) underwent contrast-enhanced MRV/CTV. Of these, 32 (76.2%) were in the CVT group, and 10 (23.8%) were in the CVT-negative group. Clinicians responsible for deciding whether additional contrast-enhanced imaging was required had access to the D-dimer results, but the neuroradiologist was blinded. Since the diagnosis was based on neuroimaging, D-dimer information did not influence the imaging process.

The brain MRI protocol included axial T1-weighted, axial and coronal T2-weighted, axial and sagittal fluid-attenuated inversion recovery (FLAIR), diffusion-weighted imaging (DWI), apparent diffusion coefficient (ADC), and susceptibility-weighted imaging (SWI) sequences. MRI examinations were performed using a 1.5 Tesla MRI scanner (General Electric Medical Systems, Milwaukee, WI, USA). The slice thickness for conventional MRI sequences was 5 mm. CT and gadolinium-enhanced imaging were not performed in pregnant patients. Gadolinium contrast agents were not used in patients with impaired renal function.

As there are no established diagnostic algorithms or specific neuroimaging parameters for CVT, each case was evaluated individually. Patients were assessed using conventional CT/CTV or MRI/MRV scans. The imaging studies were evaluated for direct visualization of the thrombus, absence of venous filling, parenchymal venous infarction, edema, hemorrhagic transformation, intracranial hypertension, hydrocephalus, and venous dilation as a vascular finding. Priority was given to looking for the “dense vessel sign,” “cord or string sign,” and “dense triangle sign” signs resulting from the direct visualization of the thrombus on thin-section CT [1]. Contrast-enhanced CTV/MRV images were specifically examined to differentiate between hypoplastic and aplastic dural sinuses. In cases of transverse sinus hypoplasia, the presence of the “notch sign” was sought on brain CT bone-window images [4].

To differentiate arachnoid granulations, features such as a typical round or oval shape, well-defined margins, and a normal flow void surrounding the granulation were sought. Imaging was performed using the MRV TOF technique and/or contrast-enhanced techniques [1].

Areas of slow flow can mimic thrombosis on CT, contrast-enhanced CT, CTV, MRV, and T1-weighted MRI. On non-contrast CT, they may appear as hyperdense vessels due to high hematocrit levels. Findings from contrast-enhanced MRV were compared with those from conventional CTV/MRV imaging to facilitate differentiation. In cases where the thrombus could not be distinguished, gradient-recalled echo or SWI techniques were used in addition to contrast-enhanced techniques [1].

All neuroimaging examinations were performed by a neuroradiologist experienced in imaging the cerebral venous system who was blinded to the quantitative D-dimer results.

### 2.5. Definition of Patient and Control Groups

Patients were divided into two groups according to radiological imaging findings. Those with findings of cerebral venous sinus thrombosis who were diagnosed with CVT were designated the CVT group. Those without findings of cerebral venous sinus thrombosis, who therefore did not receive a diagnosis of CVT, were designated the CVT-negative group.

### 2.6. Clinical and Radiological Variables in Patients with CVT

To investigate whether quantitative D-dimer levels were influenced by clinical and radiological characteristics in patients with confirmed CVT, their relationships with symptom duration, the presence and severity of neurological examination findings, the presence of parenchymal lesions, and the number of thrombosed sinuses were assessed.

Symptom duration was defined as the number of days from symptom onset to quantitative D-dimer measurement. Based on symptom duration, CVT was classified as acute (<2 days), subacute (2–14 days), or chronic (>14 days) [16]. By study design, patients with a symptom duration exceeding 30 days were not included in the analysis.

Neurological involvement was assessed using objective neurological examination findings at presentation. Impaired consciousness, motor or sensory deficits, speech impairment, cranial nerve involvement, and papilledema were considered neurological examination findings. The severity of neurological deficits at presentation was scored using the National Institutes of Health Stroke Scale (NIHSS) (assessment parameters: 1. Level of consciousness 2. Gaze 3. Visual fields 4. Facial palsy 5. Motor function (arms) 6. Motor function (legs) 7. Ataxia 8. Sensation 9. Language, 10. Dysarthria 11. Neglect). Patients with CVT presenting with isolated headache and no neurological examination findings were assigned an NIHSS score of 0 (zero).

To assess the extent of thrombotic involvement, the number of venous sinuses and veins affected on MRV was recorded. The segments assessed comprised the right and left transverse sinuses, right and left sigmoid sinuses, superior sagittal sinus, inferior sagittal sinus, straight sinus, vein of Galen, cavernous sinus, cortical veins, and right and left jugular veins. The segment length of the thrombosis and whether the occlusion was total or subtotal were not considered in this analysis. Parenchymal involvement was defined as the presence of venous infarction and/or hemorrhagic venous infarction on neuroimaging. Patients were assessed in groups with and without parenchymal lesions.

### 2.7. Data Analysis

Study data were analyzed using SPSS 31. Descriptive statistics were presented as counts, percentages, means, standard deviations, minimums, maximums, and medians. The distributional characteristics of continuous variables were assessed by jointly evaluating skewness and kurtosis values, histograms, and normal Q-Q plots. The selection of parametric and non-parametric tests was based on the distributional characteristics, sample size, and the appropriateness of the relevant test assumptions. Data distributions were also examined within each group for between-group analyses. For comparisons between two independent groups, normally distributed continuous variables were analyzed using the *t* test, and nonnormally distributed continuous variables were analyzed using the Mann–Whitney U test. Associations between categorical variables were examined using chi-square tests. Relationships between continuous variables were examined using Spearman’s rank correlation coefficient (ρ), with corresponding 95% confidence intervals (CIs). Sensitivity, specificity, positive predictive value, negative predictive value, and accuracy were calculated to assess the performance of D-dimer levels in distinguishing the diagnosis of CVT. The 95% confidence intervals for these diagnostic accuracy measures were calculated in Python (version 3.12.7) using the Math and Pandas libraries. ROC analysis was also used to assess the area under the curve (AUC), and the Youden index was used to determine the optimal cutoff.

A value of *p* < 0.05 was considered statistically significant. Effect sizes were calculated when statistically significant differences were found. For the Mann–Whitney U test, effect size (r) was classified as <0.10 (small), 0.30 (medium), and >0.50 (large). For the chi-square test, effect size ((r)) was classified as <0.10 (small), 0.30 (medium), and >0.50 (large).

## 3. Results

The records of 178 patients evaluated for suspected CVT were reviewed. A total of 81 patients meeting the exclusion criteria were excluded: those without quantitative D-dimer measurements (*n* = 70), those with insufficient clinical information (*n* = 5), those with severe infections such as sepsis, meningitis, or encephalitis (*n* = 1), and those with a symptom duration >30 (*n* = 5) (Figure 1).

The 97 patients included in the study were divided into a CVT group (*n* = 54) and a CVT-negative group (*n* = 43) based on neuroimaging findings. Diagnoses in the CVT-negative group were as follows: migraine attack without aura (*n* = 12), migraine status (*n* = 8), migraine attack with aura (*n* = 6), tension-type headache (*n* = 6), IIH (*n* = 5), new daily persistent headache (*n* = 4), brainstem migraine with aura (*n* = 1), HaNDL syndrome (headache and neurological deficits with cerebrospinal fluid lymphocytosis) (*n* = 1) (Figure 1).

Regarding demographic characteristics, 64.8% (*n* = 35) of the CVT group and 72.1% (*n* = 31) of the control group were women, with no significant difference between the groups (*p* = 0.586). The mean age was 36.67 ± 15.028 years in the CVT group and 34.56 ± 10.096 years in the control group, with no statistically significant difference between the two groups (*p* = 0.412) (Table 1).

When the groups were compared according to the quantitative D-dimer cutoff, 90.7% (49/54) of participants in the CVT group had quantitative D-dimer levels >0.5 mg/L, whereas 9.3% (5/54) had levels ≤ 0.5 mg/L. In the CVT-negative group, 95.3% (41/43) of participants had quantitative D-dimer levels ≤ 0.5 mg/L and 4.7% (2/43) had levels >0.5 mg/L. Quantitative D-dimer levels differed significantly between the two groups (*p* < 0.001) (Table 1). Of the 5 patients with false-negative results, 3 presented in the acute phase and 2 in the subacute phase; 2 patients exhibited additional neurological examination findings while 3 did not, and 1 patient had a parenchymal lesion while 4 did not. Of the two patients who received a false-positive result, one was diagnosed with tension-type headache following a COVID-19 infection, while the other was a patient with migraine attacks. All 5 patients who had symptom duration exceeding 30 days and were excluded from the study had quantitative D-dimer levels ≤ 0.5 mg/L.

Mean quantitative D-dimer levels were 1.84 ± 1.836 mg/L in the CVT group and 0.26 ± 0.147 mg/L in the control group, and the difference was significant (*p* < 0.001) (Table 1).

### Clinical and Radiological Characteristics of the CVT Group

Analysis of the timing of quantitative D-dimer testing showed that measurements were performed a mean of 6.06 ± 4.76 days after symptom onset, with a range of 1 to 21 days. Among patients with CVT, 20.4% (*n* = 11) were diagnosed in the acute phase, 70.5% (*n* = 38) in the subacute phase, and 9.3% (*n* = 5) in the chronic phase (Table 2). A statistically significant negative relationship was found between time since symptom onset and quantitative D-dimer levels (*r* = −0.301; 95% CI: −0.526 to −0.036; *p* = 0.027).

Analysis of categorical variables describing the clinical characteristics of patients in the CVT group showed that 53.7% (*n* = 29) had at least one neurological examination finding, whereas 46.3% (*n* = 25) presented to the hospital with isolated headache and no neurological examination findings (Table 2). The Mann–Whitney U test performed to examine the relationship between quantitative D-dimer levels and the presence of neurological examination findings showed no statistically significant association (Z = −1.397; *p* = 0.163). The severity of neurological examination findings was scored using the NIHSS. In patients with CVT, the mean NIHSS score was 1.76 ± 2.119, the median was 1.00, and scores ranged from 0 to 7 (Table 2). Spearman correlation analysis performed to investigate the relationship between quantitative D-dimer levels and NIHSS scores showed no statistically significant association (*r* = 0.241; *p* = 0.079).

Analysis of the number of thrombosed sinuses showed a mean of 2.26 ± 0.955, a median of 2.00, and a range of 1 to 4 (Table 2). Spearman correlation analyses were performed to determine the relationship between quantitative D-dimer levels and the number of occluded sinuses in the CVT group. No statistically significant relationship was found between quantitative D-dimer levels and the number of occluded sinuses (r = −0.068; *p* = 0.627). Regarding the proportional distribution of thrombosed sinuses and deep veins, the transverse sinuses accounted for approximately 45%, the sigmoid sinus for 26%, the sagittal sinus for approximately 16%, the internal jugular vein for 8.7%, and deep venous structures for 4.3%.

Assessment of parenchymal lesions in patients with CVT showed that lesions were present in 20.4% (*n* = 11) and absent in 79.6% (*n* = 43) (Table 2). The Mann–Whitney U test performed to determine the relationship between quantitative D-dimer levels and the presence of parenchymal lesions showed no statistically significant association (Z = −0.279; *p* = 0.780).

At a cutoff of 0.5 mg/L, quantitative D-dimer showed a sensitivity of 90.74% and specificity of 95.35% for identifying CVT. The positive predictive value was 96.08%, and the negative predictive value was 89.13%. Overall test accuracy was calculated as 92.78%. The positive likelihood ratio was 19.51, and the negative likelihood ratio was 0.10 (Table 3).

Evaluation of the diagnostic accuracy of quantitative D-dimer levels for CVT using ROC analysis yielded an AUC = 0.967 (95% CI: 0.938–0.997; *p* < 0.001), indicating high discriminatory performance for the diagnosis of CVT (Figure 2). Based on these data, the Youden index identified an optimal quantitative D-dimer cutoff of 0.495 mg/L.

Multiple linear regression analyses using sex and age were performed to identify factors affecting quantitative D-dimer levels. Sex (B = −0.115; β = −0.034; *p* = 0.742) and age (B = 0.009; β = 0.072; *p* = 0.485) had no statistically significant effect on quantitative D-dimer levels.

## 4. Discussion

In our study, quantitative D-dimer levels measured during the acute, subacute, and chronic phases were examined in 97 consecutive patients investigated for suspected CVT. At a quantitative D-dimer cutoff of >0.5 mg/L, the CVT group had statistically significantly higher quantitative D-dimer levels than the CVT-negative group. Quantitative D-dimer levels were negatively associated with symptom duration but were not affected by age, sex, the presence of neurological deficits, deficit severity, the number of thrombosed sinuses, and parenchymal damage.

Due to the retrospective design of the study, quantitative D-dimer measurement had not been performed in a large proportion (*n* = 70) of the patients (*n* = 178) whose records were reviewed. Since the diagnosis of CVT relied primarily on neuroimaging, quantitative D-dimer testing was not routinely performed. Whether or not the test was performed was not associated with the patients’ demographic, clinical, or neuroimaging characteristics; the use of the test was at the physician’s discretion.

Of the 97 patients included in the study, 54 (55.6%) were diagnosed with CVT. The high prevalence of CVT among the patients studied may be attributed to the fact that our hospital is a tertiary referral center, leading to the referral of patients with a preliminary diagnosis of CVT. Furthermore, all patients were evaluated with a preliminary diagnosis of CVT and fell into the risk group.

Headache is the first symptom in 95% of patients with CVT. The headache types reported in CVT have been described as migraine-like headache, explosive headache, chronic tension-type headache, chronic daily headache, throbbing headache, and thunderclap headache [17]. The diagnoses in our CVT-negative group (*n* = 43) were migraine attack without aura (*n* = 12), migraine status (*n* = 8), migraine attack with aura (n = 6), tension-type headache *(n* = 6), IIH (*n* = 5), new daily persistent headache (n = 4), brainstem migraine with aura (*n* = 1), and HaNDL syndrome (*n* = 1). The headache types described in CVT were consistent with the diagnoses in our differential diagnosis. HaNDL syndrome can also mimic CVT through neurological examination findings such as migraine-type headache, transient neurological deficits, and papilledema [18]. The underlying cause of a headache is sometimes unknown, and neuroimaging can sometimes be vital even in the absence of focal neurological signs.

In our study, 64.8% of the patients with CVT were female and 35.2% were male; 72.1% of the control group were female and 27.9% were male, with no statistically significant difference in sex between the groups (*p* = 0.586). The mean age was 36.67 ± 15.02 in the CVT group and 34.56 ± 10.09 in the control group, and the difference was not statistically significant (*p* = 0,412). The literature has reported that CVT is more common in women, with a mean proportion of women of 73% and a mean age of 36.86 (±13.7) years [19,20]. Our findings are consistent with previous research reports. Unlike arterial stroke, CVT is a disease of young people. Its greater frequency in women may be attributable to sex-specific risk factors. Studies have shown that CVT is closely associated with estrogen-containing oral contraceptives, hypercoagulability during pregnancy and the postpartum period, infections, blood loss during childbirth, dehydration, and fluctuations in intracranial pressure during childbirth [21].

At a quantitative D-dimer cutoff of >0.5 mg/L, 90.7% (49/54) of patients with CVT tested positive (D-dimer level > 0.5 mg/L), whereas 9.3% (5/54) tested negative (D-dimer level ≤ 0.5 mg/L). Among CVT-negative participants, 95.3% (41/43) tested negative (quantitative D-dimer level ≤ 0.5 mg/L), and 4.7% (2/43) tested positive (quantitative D-dimer level > 0.5 mg/L). In a published study including 73 patients with confirmed CVT, D-dimer levels were within normal limits in 10% of the patients [22]. Another study, which excluded pregnant and postpartum patients and those with thrombophilia or infections, detected elevated D-dimer levels in 80% of patients with CVT [23]. In another study with a methodology similar to ours, D-dimer levels were elevated in 97% of patients with CVT and 8.7% of those without CVT; our findings are consistent with the literature [15]. Pregnancy, the postpartum period, malignancy, or other inflammatory processes may have increased quantitative D-dimer levels in those without CVT, resulting in false-positive results [24]. Dentali et al. reported isolated headache, longer symptom duration, and limited sinus involvement as potential risk factors for false-negative D-dimer results in patients with CVT [25]. The mean quantitative D-dimer level was 1.84 ± 1.836 in the CVT group and 0.26 ± 0.147 in the control group, and the difference was statistically significant (*p* < 0.001). Studies have also shown that the mean D-dimer level is significantly higher in patients with CVT than in those without CVT (0.876 mg/L versus 0.538 mg/L) [26]. In another similar study, mean D-dimer levels were significantly higher in patients diagnosed with CVT (2.052 ± 1.286 mg/L) than in those without CVT (0.375 ± 0.368 mg/L) [15]. Our findings are consistent with the literature.

In our study, quantitative D-dimer showed a sensitivity of 90.7% and a specificity of 95.4% for identifying CVT. Its positive predictive value was 96.1%, and its negative predictive value was 89.1%. The overall accuracy of the test was 92.8%, the positive likelihood ratio was 19.51, and the negative likelihood ratio was 0.10. ROC analysis yielded an AUC = 0.967 (95% CI: 0.938–0.997; *p* < 0.001) for quantitative D-dimer. In one similar study of consecutive patients with suspected CVT who had isolated headache, a normal neurological examination, and a normal standard brain CT, quantitative D-dimer had a sensitivity of 97.8%, a specificity of 84.9%, a positive predictive value of 33%, and a negative predictive value of 99.8% [10]. A recent study reported a negative predictive value of 96% for the high-probability CVT group and 99% for the combined low- and intermediate-probability group [19]. Differences in the percentages across studies may be attributable to differences in study methodology, patient selection, group characteristics, or symptom duration. The diagnosis of CVT is primarily based on MRI/MRV or CT/CTV [1]. Although quantitative D-dimer cannot replace neuroimaging as a diagnostic test, it can be used as a supportive diagnostic tool for CVT in selected patients.

In our study, clinical and radiological presentations at hospital admission were assessed to investigate whether quantitative D-dimer levels reflected clinical and radiological characteristics.

Based on the time from symptom onset to hospital presentation, 20.4% of the patients were diagnosed in the acute phase, 70.5% in the subacute phase, and 9.3% in the chronic phase. Correlation analyses revealed a statistically significant negative association between symptom duration and quantitative D-dimer levels (*p* = 0.027). CVT was diagnosed after a mean of approximately 6.1 (1–21) days. Other studies have also shown a negative correlation between D-dimer and symptom duration, with elevated D-dimer levels associated with acute symptom onset [16]. In venous thromboembolism, D-dimer levels typically reach 25% of the initial value after 1–2 weeks. This raises the possibility that patients presenting with subacute or chronic symptoms may have negative D-dimer results [27]. As expected, unlike arterial stroke, CVT is most often diagnosed in the subacute or chronic phase [28]. The time from symptom onset to hospital presentation in patients with CVT has generally been reported to be less than seven days [29]. Our findings are consistent with the literature. Delayed diagnosis of CVT may be due to the fact that parenchymal damage does not always occur and neurological deficits are therefore absent, delayed differential diagnosis in patients with isolated headache, the late development of occlusion due to gradual thrombus growth, or compensation for the occluded sinus by collateral vessels.

At hospital presentation, at least one neurological examination finding was identified in 53.7% of patients with CVT; 46.3% had isolated headache with no additional neurological examination findings. The Mann–Whitney U test performed to investigate the relationship between quantitative D-dimer levels and the presence of neurological examination findings revealed no statistically significant correlation (*p* > 0.05). NIHSS scores were calculated for each patient to assess the relationship between quantitative D-dimer levels and the severity of neurological examination findings. Correlation analyses revealed no significant association between patients’ NIHSS scores and quantitative D-dimer levels (*r* = 0.241; *p* = 0.079). Published studies and meta-analyses have reported no neurological examination findings in 47.7% of patients with CVT and focal neurological deficits in 20–54% [10,28]. Our results are consistent with both findings in the literature. Similar to our study, some published studies have found no significant association between the presence of focal neurological deficits and D-dimer levels [15]. Other studies, however, have used a clinical scoring system developed by the investigators themselves and reported that quantitative D-dimer levels increased in parallel with clinical status regarding the presence and severity of CVT-related neurological deficits [24]. A study using the modified Rankin Scale reported significant associations between D-dimer levels at hospital admission and neurological functional impairment at discharge in patients with CVT [30]. These differences in findings may be due to differences in the scales used to measure neurological deficits. The location and extent of cerebral lesions may also have affected the findings. Individual variations in venous collateralization may also have influenced the presence or severity of neurological examination findings. The absence of a statistically significant relationship between quantitative D-dimer and NIHSS may be attributed to the relatively small sample size. Early diagnosis of CVT in patients with isolated headache and no neurological deficits is difficult, is often delayed, and requires a high level of clinical awareness. Nevertheless, elevated D-dimer levels in these patients and the fact that these levels were unaffected by the presence of neurological deficits are diagnostically important.

Analyses of the number of thrombosed sinuses in patients with CVT showed that the number ranged from 1 to 4, with a mean of 2.26 ± 0.955 occluded sinuses. Regarding the proportional distribution of thrombosed sinuses and deep veins, the figures were approximately 45% for the transverse sinuses, 26% for the sigmoid sinus, approximately 16% for the sagittal sinus, 8.7% for the internal jugular vein, and 4.3% for deep venous structures; these findings were consistent with the literature [1]. Spearman correlation analyses performed to determine the relationship between quantitative D-dimer levels and the number of thrombosed sinuses revealed no statistically significant association (*p* = 0.627). Published studies have found elevated D-dimer levels to be associated with thrombosis in more than one sinus [15,16]. Our findings differ from those in the literature. Although a greater number of thrombosed sinuses would be expected to lead to greater fibrin degradation and dissemination, the number of occluded sinuses may not have fully reflected the thrombus burden. Thus, differences in findings may be due to qualitative rather than quantitative features, such as the location of the thrombosed sinus, whether the occlusion is total or subtotal, and the length of the involved segment. The timing of quantitative D-dimer analysis and patient characteristics may also have influenced the findings. The absence of a statistically significant relationship between quantitative D-dimer levels and the number of thrombosed sinuses may be attributed to the relatively small sample size.

Analyses of parenchymal damage in patients with CVT showed that 79.6% had no parenchymal damage, whereas 20.4% had infarction and/or hemorrhagic infarction. Subarachnoid hemorrhage was additionally observed in 2 patients (3.7%) with hemorrhagic infarction. Mann–Whitney U test analyses performed to determine the relationship between quantitative D-dimer levels and the presence of parenchymal lesions revealed no statistically significant correlation (*p* > 0.05). The mechanism of parenchymal damage in CVT is attributable to impaired drainage of blood into the venous return system. Blood that cannot drain through veins and sinuses occluded by thrombosis increases hydrostatic pressure, and cerebral edema develops in the affected region. Capillary hemorrhages due to elevated pressure lead to hemorrhagic infarctions, which are highly characteristic of CVT. Bleeding may also extend into the adjacent subarachnoid space, resulting in subarachnoid hemorrhage [31]. The absence of a statistically significant relationship between quantitative D-dimer levels and the presence of parenchymal lesions may be attributed to the relatively small sample size.

Multiple linear regression analyses including sex and age as variables were performed to identify factors affecting quantitative D-dimer levels. Neither sex (B = −0.115; β = −0.034; *p* = 0.742) nor age (B = 0.009; β = 0.072; *p* = 0.485) had a statistically significant effect on quantitative D-dimer levels. Similar studies have also reported that age and sex do not affect D-dimer levels and that there is no significant association between them [15].

Using the Youden index and our study data from 97 patients, the optimal cutoff for quantitative D-dimer was determined to be 0.495 mg/L. Our result is highly consistent with the quantitative D-dimer cutoff accepted and used in the literature [15].

Limitations: Although quantitative D-dimer levels achieved high predictive values for diagnosing or ruling out CVT in our study, the greatest limitation of the test is the lack of specificity of elevated blood levels. Given that pregnancy, the postpartum period, infections, and malignancies are among the etiological factors for CVT, elevated quantitative D-dimer levels may also be attributable to one of these coexisting conditions. Because our study was retrospective, height and weight records were not available for every patient, and the relationship between body mass index and quantitative D-dimer levels could not be examined. The sample in this article is relatively small and single-center; therefore, the results cannot be generalized.

Strengths: Our study is based on real-world data. The likelihood of false-negative or false-positive results was reduced by excluding patients with symptom durations exceeding 30 days and those with overt infections such as meningitis, encephalitis, or sepsis. MRI evaluations were performed by a neuroradiologist with extensive experience in the field who was blinded to the quantitative D-dimer results. The fact that the neuroradiologist was blinded to the D-dimer results reduced the risk of reference standard interpretation bias.

Conclusion: Elevated quantitative D-dimer values alert us to CVT, particularly in patients with isolated headache and no neurological deficits. The diagnosis of CVT is primarily based on MRI/MRV or CT/CTV, and high quantitative D-dimer levels may support the diagnosis.

## Figures and Tables

**Figure 1:**
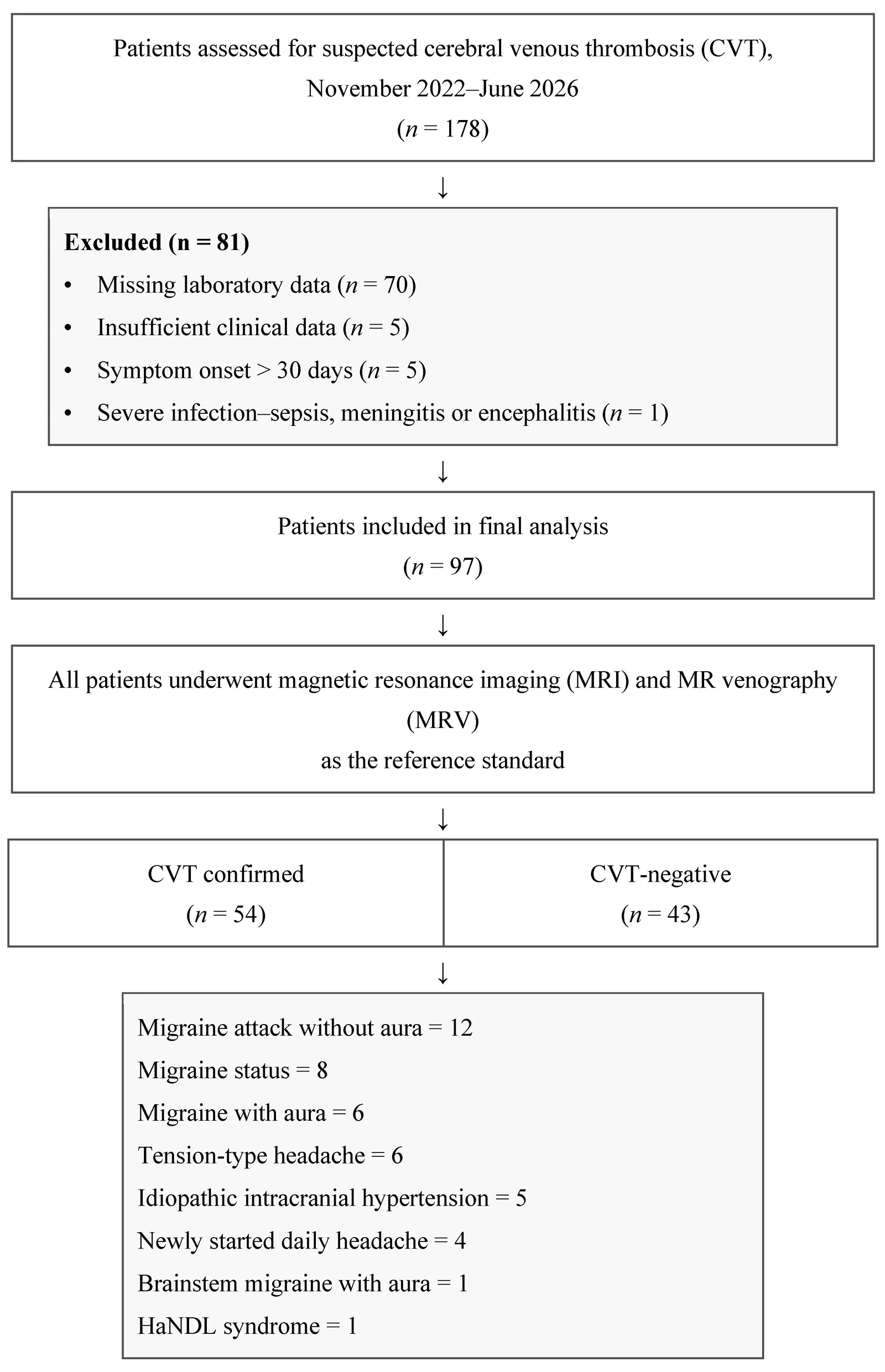
STARD flow diagram of patient assessment, inclusion, and diagnostic classification for suspected cerebral venous thrombosis (CVT).

**Figure 2:**
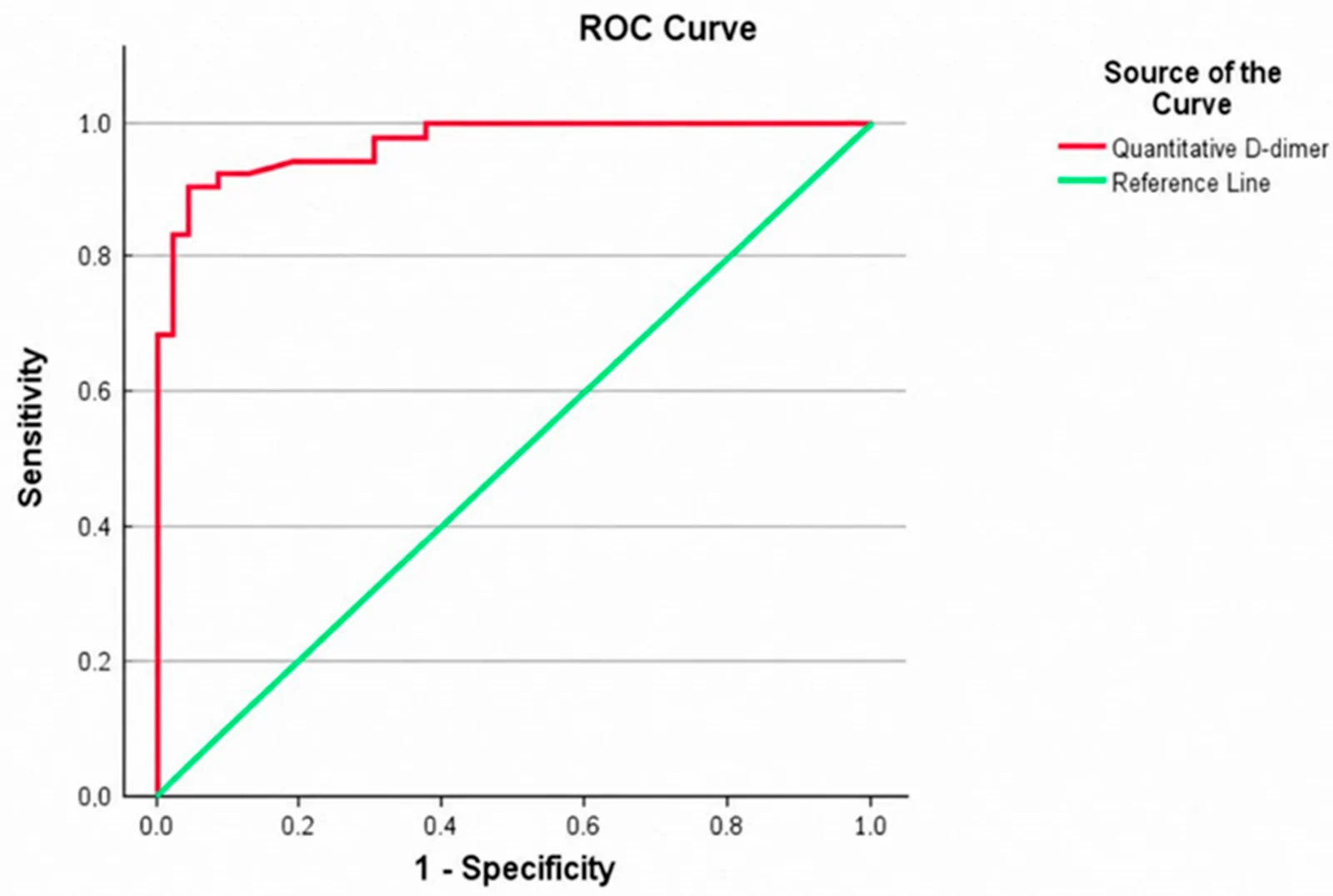
ROC Analysis Curve.

**Table 1:** Analyses of the Sociodemographic and Clinical Characteristics of the Groups.

Variables		CVT-Negative (*n* = 43)		CVT (*n* = 54)		t/Z/X^2^	*p*	d/r
		*n*	**%**	*n*	**%**			
Gender	Female	31	72.1	35	64.8	0.296 ^1^	0.586	0.055
	Male	12	27.9	19	35.2			
Quantitative D-dimer	≤0.5 mg/L	41	95.3	5	9.3	67.744 ^1^	<0.001	0.836
	>0.5 mg/L	2	4.7	49	90.7			
		Mean ± SD	Median (min-max)	Mean ± SD	Median (min-max)			
Age (year)		34.56 ± 10.096	34.00 (18–60)	36.67 ± 15.028	32.00 (18–72)	−0.824 ^2^	0.412	−0.161
Quantitative D-dimer mg/L		0.26 ± 0.147	0.20 (0.07–0.83)	1.84 ± 1.836	1.01 (0.24–7.70)	−7.901 ^3^	<0.001	−0.802

^1^ Chi Square Test, ^2^ *t*-test, ^3^ Mann–Whitney U Test; CVT: Cerebral venous thrombosis; SD: Standard deviation, Min-Max: Minimum-maximum.

**Table 2:** Descriptive Findings Regarding Clinical Characteristics in the CVT Group.

Variables		Mean ± SD	Median (Min–Max)
Duration of symptoms		6.06 ± 4.768	4.50 (1–21)
NIHSS Score		1.76 ± 2.119	1.00 (0–7)
Number of thrombosed sinuses		2.26 ± 0.955	2.00 (1–4)
		*n*	%
Neurological examination finding	No	25	46.3
	Yes	29	53.7
Parenchymal lesion	No	43	79.6
	Yes	11	20.4
Time periods	Acute	11	20.4
	Subacute	38	70.5
	Chronic	5	9.3

CVT: Cerebral venous thrombosis; SD: Standard deviation, Min-Max: Minimum-maximum.

**Table 3:** D-Dimer values of the groups and diagnostic accuracy results for CVT.

Quantitative D-Dimer	CVT-Negative (*n* = 43)		CVT (*n* = 54)	
	*n*	**%**	*n*	**%**
>0.5 mg/L	2	4.7	49	90.7
≤0.5 mg/L	41	95.3	5	9.3
Diagnostic Accuracy Indicators		Value (%95 CI)		
Sensitivity (%)		90.74 (80.09–95.98)		
Specificity (%)		95.35 (84.54–98.72)		
Positive Predictive Value (%)		96.08 (86.78–98.92)		
Negative Predictive Value (%)		89.13 (76.96–95.27)		
Accuracy Rate (%)		92.78 (85.85–96.46)		
Positive Probability Ratio		19.51 (5,03–75.71)		
Negative Probability Ratio		0.10 (0.04–0.22)		

CI: Confidence interval; CVT: Cerebral venous thrombosis.

## Data Availability

The data used in this study are available from the corresponding author upon request.
